# Match play performance characteristics that predict post-match creatine kinase responses in professional rugby union players

**DOI:** 10.1186/2052-1847-6-38

**Published:** 2014-11-03

**Authors:** Marc R Jones, Daniel J West, Bradley J Harrington, Christian J Cook, Richard M Bracken, David A Shearer, Liam P Kilduff

**Affiliations:** Applied Sports Technology Exercise and Medicine Research Centre (A-STEM), Health and Sport Portfolio, Talbot Building, College of Engineering, Swansea University, Singleton Park, Wales, SA2 8PP UK; Scarlets Rugby, Llanelli, SA14 9UZ UK; Department of Sport, Exercise & Rehabilitation, Faculty of Health and Life Science, Northumbria University, Northumberland Building, Newcastle upon Tyne, NE1 8ST UK; School of Sport, Health and Exercise Science Bangor University, Bangor, Gwynedd, LL572PZ Wales; Faculty of Life Sciences and Education, University of South Wales, Swansea, CF37 1DL Wales

**Keywords:** Athlete management, Performance analysis, Muscle damage

## Abstract

**Background:**

Rugby union players can take several days to fully recover from competition. Muscle damage induced during the match has a major role in player recovery; however the specific characteristics of match play that predict post-match muscle damage remains unclear. We examined the relationships between a marker of muscle damage and performance characteristics associated with physical contacts and high-speed movement in professional rugby union players.

**Methods:**

Twenty-eight professional rugby union players (15 forwards, 13 backs) participated in this study. Data were obtained from 4 European Cup games, with blood samples collected 2 h pre, and 16 and 40 h post-match, and were subsequently analysed for creatine kinase (CK). Relationships between changes in CK concentrations and number of physical contacts and high-speed running markers, derived from performance analysis and global positioning system (GPS) data, were assessed.

**Results:**

Moderate and moderate-large effect-size correlations were identified between contact statistics from performance analysis and changes in CK at 16 and 40 h post-match in forwards and backs, respectively (*e.g.* backs; total impacts vs. ΔCK (r = 0.638, p < 0.01) and Δ% CK (r = 0.454, p < 0.05) 40 h post-match). Furthermore, moderate effect-size correlations were found between measures of high-speed running and sprinting, and changes in CK at 16 and 40 h post-match within the backs (e.g. high-speed running distance vs. ΔCK (r = 0.434, p = 0.056) and Δ% CK (r = 0.437, p = 0.054) 40 hrs post-match).

**Conclusions:**

Our data demonstrate that muscle damage induced by professional rugby union match play is to some extent predicted by the number of physical contacts induced during performance. Furthermore, we show for the first time that muscle damage in backs players is predicted by high-speed running measures derived from GPS. These data increase the understanding of the causes of muscle damage in rugby union; performance markers could potentially be used to tailor individual recovery strategies and subsequent training following rugby union competition.

## Background

Rugby union is a physically demanding game characterised by repeated high-intensity bouts of relatively short duration exercise, with varying recovery periods [1, 2]. The high-intensity bout characteristics are largely positional dependent [1]; however, all players are exposed to a high magnitude of physical contacts and collisions [3, 4], and high intensity stretch-shortening cycle (SSC) movements. Research has demonstrated large elevations in blood markers of muscle damage (e.g. creatine kinase) [3, 5, 6], and disruptions to neuromuscular [7], hormonal [3, 7, 8], immune functions [3] and mood [7] for several days following competition. As a consequence, players may take several days to fully recover following competition [7]. Insufficient recovery following competition may compromise players’ ability to train, and with the accumulative stress of subsequent training, may compromise preparation for subsequent games [9]. Furthermore, acute and chronic insufficient recovery may predispose an individual to a greater risk of injury [10] and the development of overtraining syndrome [11]. Therefore insufficient recovery has significant implications for player preparation and performance.

Previous research has also highlighted the individual nature of recovery in professional rugby union [7]. For example, West et al. [7] found countermovement peak power output of ~58% of players had not returned to pre-match values 60 h post-match. Knowledge of individual recovery patterns could therefore be used to benefit player management through individual modification of subsequent training and recovery strategies [7]. However, assessing recovery following each game is often associated with invasive collection procedures and analysis which may take several hours or days to conduct (e.g. hormone analysis from saliva sample, [12]; muscle damage from blood sample, [3]). Consequently this information is often not available in time to assess individual recovery and subsequent weekly training modulation. It has been suggested that match play performance characteristics could be used to prospectively predict individual recovery in rugby union [3, 5, 6] and other team sports [12–14]. Physical contacts are known to predict post-match creatine kinase (CK; an indirect blood marker of muscle damage) responses in rugby union [3, 5, 6] and neuromuscular recovery in rugby league [13], suggesting recovery may be determined by the extent of mechanical damage induced through contact during performance in both rugby codes.

With the metabolic, mechanical and neural elements associated with fatigue following SSC activity [15], it is also possible that muscle damage and neuromuscular fatigue following rugby union may partly be determined by the extent of high-intensity movement characteristics. Indeed, through advances in match analysis technologies, global positioning system (GPS) tracking has shown relationships between high-speed running characteristics and changes in CK [12, 16] and neuromuscular function [17] during the recovery period in team sports, such as soccer [12] and Australian football [16], however at present this analysis has not been carried out in professional rugby union. Therefore, the aim of this study was to examine if performance characteristics associated with high-speed movements and physical contacts during match play can predict post-match changes in creatine kinase, a surrogate marker of muscle damage, in professional rugby union players.

## Methods

### Methods overview

Performance characteristics and the subsequent recovery process were assessed following four group stage matches of the 2012-2013 European Cup, the elite competition in Northern Hemisphere club rugby. Players were required to report to testing 2 h prior to kick off and approximately 16 and 40 h following the end of the match. On each testing occasion a finger-prick blood sample was collected for the assessment of creatine kinase (CK); a blood marker of muscle damage. Players were separated into forwards and backs groups to assess the correlation between pre to post-match changes in CK and performance characteristics derived using global positioning system (GPS) and notational analysis.

### Subjects

Thirty-six male rugby union players volunteered for the study. Participant data files were only included in the analysis if they performed a minimum of 60 min of match play [13, 14]. Subsequently, only data from 28 players (age 25.1 ± 3.1 yrs) were analysed (15 forwards, 26.7 ± 2.8 yrs, 111.6 ± 5.7 kg; 13 backs, 23.4 ± 2.6 yrs, 94.2 ± 7.9 kg).

### Procedures

Ethical approval was given by the Swansea University Ethics Committee. Prior to providing informed consent, participants were given information outlining the rationale, potential applications of the study and procedures. Upon arrival on testing days, players placed their non-dominant hand in a basin of warm water (40-42°C) for 2 min prior to capillary blood collection. Immediately upon removal from the water, a pinprick on the finger of the warmed hand was made using a spring-loaded single use disposable lancet (Accu-Chek Safe-T-Pro Plus; Roche, Basle, Switzerland) which allowed approximately 300 μl of capillary whole blood to be collected (Microcuvette® CB300, Sarstedt, Numbrecht, Germany). Samples were then put on ice until being centrifuged for 10 min at 3000 rpm (Heraeus Sepatech Labofuge 200; Kendro Laboratory Products, Germany), from which plasma was pipetted into an eppendorf tube and stored at -70°C until analysis. This process was repeated at 16 and 40 h post-match, the time points at which the players were returning to structured training post-match [3]. All samples were taken at the same time of day and players were not permitted to carry out any other training between sample points (i.e. they were at rest). Plasma was analysed for CK using an automated clinical chemistry analyser (Cobas Miras; ABX diagnostics, Beds., UK). The intra-assay coefficient of variance (CV) was 0.93 ± 0.00%.

Global positioning system units (10Hz; MinimaxX v.4.0, Catapult Innovations, Melbourne, Australia) were fitted into the back of a custom-made vest so that the unit was positioned in the centre area of the upper back and slightly superior to the shoulder blades, with no restriction to the range of movement of the upper limbs and torso. GPS data was downloaded and analysed using Catapult Sprint software (Catapult Innovations, Melbourne, Australia) for analysis of distances covered. Distances at different velocity zones were analysed according to the zone classification system provided by McLellan et al. [18], with further separation of low and high-speed movement in accordance with that described by Gabbett et al. [19]. High-speed measures analysed were high-speed running distance (>5 m.s^-1^), sprint distance (>5.6 m.s^-1^) and number. The units were also used to provide total contacts from performance. Tackle detection; a feature of the MinimaxX unit (Catapult Innovations, Melbourne, Australia) which incorporates a tri-axial piezoelectric linear accelerometer system, gyroscopes and magnetometers imbedded within the unit has been shown to provide a valid quantification of the contact load in rugby league [20]. In addition, the nature of physical contacts were also analysed by the teams’ performance analyst. Specifically, Sportscode (Sportstec, NSW) was used to provide frequency counts for the number of tackles, hit ups, and player contacts at the breakdown area, providing a measure of total impacts comparable to Smart et al. [5]. Finally, the number of scrums engaged by forwards were also analysed.

Immediately post-game players followed a standardised recovery protocol and were not permitted to engage in any physical training. The recovery protocol entailed contrast water therapy (2 min warm (40-42°C), 2 min cold (10-15°C) ×3), with players finishing in cold water. Following the next days testing (approx. 16 h post-match), players then performed an active recovery session that involved 3 sets of 4 min bike or cross-trainer at 65 ± 5% age predicted max heart rate, followed by 4 min light movement on the same equipment.

### Statistical analyses

Analysis of movement and contact characteristics and their correlations with changes in CK were conducted within the forwards (props, hooker, second rows, flankers and number 8) and backs (scrum-half, outside-half, centres, wingers and full back) positional groups due to movement and contact variances between groups [4]. To identify and remove outliers from the data, Z-scores for CK at each time points were analysed. The criterion for an outlier at a time point was defined as any value greater than 2 standard deviations from the position mean [21].

Global positioning system data analysed using Catapult Sprint software (Catapult Innovations, Melbourne, Australia) were exported to CSV files for further analysis in Microsoft Excel (Microsoft Office Excel 2010, Microsoft Corporation, Berkshire, UK). A mixed model ANOVA was used to assess changes in CK concentrations from pre-game to post-game and assess movement and contact statistics within each positional group. Significant differences in CK concentrations were located by a Bonferroni corrected pair-wise comparisons. The criterion level for significance was set at p < 0.05. Correlations between CK and performance characteristics within each group were analysed using the Pearson Product-Moment Correlation Coefficient. Magnitudes of effect of the correlations were determined as follows: trivial <0.10; small ≤0.10-0.29; moderate 0.30-0.49; large 0.50-0.69; very large 0.70-0.89; and nearly perfect 0.90-0.99 [22]. All statistical analyses were performed using the Statistical Package for the Social Sciences (SPSS for Windows, version 14.0; SPSS, Inc., Chicago, IL, USA).

## Results

In total 57 data cases (45 including GPS) were collected with each forward and back playing an average of 2.0 ± 0.6 and 2.1 ± 1.0 games each. When separated into position groups, 27 backs (22 including GPS) and 30 forwards (23 including GPS) data files were used for analysis.

Table 1 shows positional differences between forwards and backs. Results demonstrate that contact demands were significantly greater for forwards than backs. However, backs, played significantly longer than forwards (p < 0.05), covered greater total distances (p < 0.01), covered greater distance per minute (m.min^-1^; p < 0.01), performed a greater number of sprints (p < 0.001), and covered greater distances sprinting and at high-speed (p < 0.001; Table 1).

**Table 1:** **Summary of performance markers derived from global positioning system (GPS) and performance analysis data**

	Forwards	Backs
*Performance analysis*	*n = 30*	*n = 27*
Game time (min)	80 ± 13	87 ± 11^a^
Tackles	5 ± 3	4 ± 3
Hit-ups	5 ± 2	5 ± 3
Contacts hit	15 ± 6^c^	6 ± 4
Total impacts	25 ± 9^c^	15 ± 7
Scrum number	13 ± 5^c^	0 ± 0
*GPS*	*n = 23*	*n = 22*
Distance (m)	4906 ± 902	5959 ± 1013^b^
m.min^-1^	60.4 ± 7.8	67.8 ± 8.2^b^
Sprint number	7 ± 6	18 ± 6^c^
Sprinting (m)	121 ± 112	333 ± 122^c^
High-speed running (m)	231 ± 167	509 ± 150^c^
Total contacts	31 ± 14^c^	16 ± 7

Value significantly greater than other positional group ^a^< 0.05 ^b^< 0.01 ^c^< 0.001.

m metres; m.min^-1^ metres per min; min minutes.

Data presented as mean ± SD.

There was a significant time effect (p < 0.05) and a significant effect of player positional group (p < 0.05) in the plasma CK responses. Baseline plasma CK concentrations were similar between backs and forwards (274 ± 155 and 368 ± 127 IU.L^-1^; p < 0.01), however CK concentrations were significantly increased at all post-match time points (p < 0.05), with changes in CK greater in backs, when compared to forwards at 16 h (forwards Δ705 ± 483 vs. backs Δ1237 ± 871 IU.L^-1^; p < 0.01) but not at 40 h post-match (forwards Δ383 ± 329 vs. backs Δ540 ± 412 IU.L^-1^, p = 0.12).

Moderate to large effect size correlations between changes in CK and performance analysis and GPS measures from match play are displayed in Tables 2 and 3, respectively.

**Table 2:** **Correlation between changes in creatine kinase (CK) and performance analysis characteristics**

	Forwards (n = 30)				Backs (n = 27)			
	ΔCK		Δ% CK		ΔCK		Δ% CK	
	+16 hrs	+40 hrs	+16 hrs	+40 hrs	+16 hrs	+40 hrs	+16 hrs	+40 hrs
Tackles	0.230	0.275	0.331	0.305	0.068	0.579**	0.025	0.386
	Small	Small	Moderate	Moderate	Trivial	Large	Trivial	Moderate
Hit-ups	0.448*	0.406*	0.494**	0.387*	0.407*	0.201	0.309	0.061
	Moderate	Moderate	Moderate	Moderate	Moderate	Small	Moderate	Trivial
Contacts hit	0.188	0.097	0.274	0.188	0.395	0.518**	0.463*	0.444*
	Small	Trivial	Small	Small	Moderate	Large	Moderate	Moderate
Total impacts	0.330	0.277	0.438*	0.343	0.404	0.638**	0.388	0.454*
	Moderate	Small	Moderate	Moderate	Moderate	Large	Moderate	Moderate
Scrum number	0.186	0.183	0.232	0.219				
	Small	Small	Small	Small				

Level of significance *p < 0.05; **p < 0.01.

Effect Sizes <0.10 trivial, 0.10-0.29 small, 0.30-0.49 moderate, 0.50-0.69 large, 0.70-0.89 very large, 0.90-0.99 nearly perfect.

ΔCK change in creatine kinase; Δ% CK percentage change in creatine kinase.

**Table 3:** **Correlation between changes in creatine kinase (CK) and movement and performance markers derived from global positioning system (GPS) data**

	Forwards (n = 23)				Backs (n = 22)			
	ΔCK		Δ% CK		ΔCK		Δ% CK	
	+16 hrs	+40 hrs	+16 hrs	+40 hrs	+16 hrs	+40 hrs	+16 hrs	+40 hrs
Sprint number	-0.172	-0.132	-0.149	-0.102	0.339*	0.419*	0.364*	0.332*
	Small	Small	Small	Small	Moderate	Moderate	Moderate	Moderate
Sprinting (m)	-0.118	-0.060	-0.073	-0.016	0.409*	0.420*	0.447*	0.359*
	Small	Trivial	Trivial	Trivial	Moderate	Moderate	Moderate	Moderate
High-speed (m)	-0.195	-0.133	-0.139	-0.079	0.239	0.434*	0.346*	0.437*
	Small	Small	Small	Trivial	Small	Moderate	Moderate	Moderate
Total contacts	-0.204	-0.309	-0.146	-0.238	-0.097	0.113	0.080	0.363*
	Small	Moderate	Small	Small	Trivial	Small	Trivial	Moderate

Level of significance *p < 0.05.

Effect Sizes <0.10 trivial, 0.10-0.29 small, 0.30-0.49 moderate, 0.50-0.69 large, 0.70-0.89 very large, 0.90-0.99 nearly perfect.

ΔCK change in creatine kinase; Δ% CK percentage change in creatine kinase; m metres.

Analysis of forwards show significant moderate correlations between various contact measures derived from performance analysis and both ΔCK and % change in CK (Δ% CK) at 16 and 40 h post-match (Table 2, p < 0.05).

Significant moderate to large correlations were observed for the backs between contact measures derived from performance analysis and both ΔCK and Δ% CK at 16 and 40 h post-match (p < 0.05). Furthermore, non-significant, moderate correlations were observed between GPS measures associated with high-speed movement and both ΔCK and Δ% CK at 16 and 40 h post-match (Table 3).

No significant correlations were found between total contacts detected by micro-sensors within the GPS units and ΔCK, however a moderate effect size correlation was observed with Δ% CK at 40 h post-match for the backs (Table 3).

## Discussion

The aim of this study was to investigate the relationships between performance markers associated with physical contacts and high-speed movement, and post-match changes in creatine kinase (CK) in professional rugby union players. Here we demonstrate that muscle damage, as indicated by CK concentrations, is to some extent predicted by the number of physical contacts performed during rugby union match play. Furthermore, we show for the first time that the muscle damage experienced by backs is associated with high-speed running derived from GPS.

In accordance with previous research [3], maximum CK levels were observed at 16 h post-match, with values remaining significantly elevated from pre-game values at 40 h post-match. Furthermore, we demonstrated significant relationships between various contact measures derived from performance analysis (e.g. tackles made, hit-ups) and changes in CK concentrations at both 16 and 40 h post-match. These data are consistent with previous research demonstrating that increases in muscle damage from rugby union match play are largely determined by the extent of mechanical damage induced through physical contact [3, 5, 6], with body to body contacts, collisions and tackles being significantly related to changes in CK concentrations [3]. These findings may be explained by the blunt force trauma from physical contacts which disrupt skeletal tissue structure integrity; subsequently increasing cell permeability and the diffusion of soluble enzymes such as CK into the interstitial fluid [23].

The present study is the first to demonstrate that muscle damage induced by match play is to some extent correlated with high-speed movement characteristics in rugby union backs. Here we demonstrate moderate effect size correlations between sprint number, sprint distance, and high-speed running distance with ΔCK and Δ% CK at 16 and 40 h post-match for backs. These relationships may be explained by high-force, eccentric work when performing SSC activities such as high-speed running This force may exceed the muscles ability to actively resist load, forcing the muscle to lengthen and generate greater active tension [24]. Therefore, in addition to monitoring the extent of contact performed, monitoring the extent of high-speed running conducted may be important in attempting to predict the extent of muscle damage induced from match play in back players.

The relationships between movement characteristics and changes in CK concentrations found in the present study support previous research which show correlations between measures related to high intensity running distance (>5 m.s^-1^) and an increase in CK levels in semi-professional football [12], however, we are the first to demonstrate this in professional rugby union. Thorpe and Sunderland [12] found that the change in ΔCK was related to the total number of sprints performed (r = 0.80) and sprint distance (r = 0.78), while percentage change in %ΔCK correlated to sprint number (r = 0.86), sprint distance (r = 0.89) and high intensity distance covered (>4.167 m.s^-1^; r = 0.92). Furthermore in Australian rules football, reductions in neuromuscular function and changes in CK post-match have been demonstrated to have stronger correlations with measures associated with high-speed running than total running distance [16], suggesting that muscle damage and neuromuscular recovery following competition in rugby union is more likely determined by the extent of high-intensity SSC activity performed than total distance covered. Although we did not measure neuromuscular function this should not detract from the application of our data, as previous research has demonstrated strong associations between functional recovery and blood markers of muscle damage [25, 26].

Although correlation is by no means causation, a potential explanation for the relationships between high-speed movement and changes in CK only being observed in backs is likely due to their greater high-speed running demands when compared to the forwards [1, 2, 4]. Additionally, it may also be due to the velocity thresholds used in the present study. Dwyer and Gabbett [27] identify that many sprints in team sport involve maximal efforts of a short duration (~1-2 s) which do not allow the attainment of high-speed and are thus not reported as high-speed or sprinting efforts. Previous research has demonstrated that changes in CK concentration following Australian rules football were correlated to high acceleration metres covered [16], thus correlations for forwards and backs may have been stronger in the present study if high-acceleration movements were included in analysis in addition to high-speed movement.

Previous research has demonstrated that forwards spend a greater duration in static exertion than backs [2]. However, with only moderate correlations found between contact statistics and muscle damage in the present study for forwards, quantifying the extent of contact may be important in determining the extent of work done. For example, in the present study small effect size correlations were found between scrum number and changes in CK. Smart et al. [5] describe that at scrum engagement, the large impact created from the momentum of both packs is likely to create substantial physical trauma and impact in the front row, however the impact and trauma experienced by back row forwards; who play a ‘supporting role’, is likely to be less substantial. Therefore although contact statistics may give an indication of the extent of muscle damage experienced by players, it does not account for the extent of contact or exertion. Furthermore, in the present study, total contacts detected within the GPS units only showed a moderate correlation with %ΔCK within the backs at 40 h post-match. Thus, despite advances in GPS technology in quantifying physical contacts, further research is required.

We were limited in our ability to perform additional measures alongside the measurements of CK, such as neuromuscular function and hormonal responses due to limited time and facilities available for testing prior to each match. Indeed, it would be important to examine the extent performance characteristics may predict changes in these measures post-match as there is potential for neuromuscular function reduction to remain, despite normalised CK [28], and steroid hormone profiles [7]. However, despite these limitations the results of this study add to the literature and our understanding of post-match changes in muscle following rugby union match play and we believe our data have implications for player management post-match. As an alternative to assessing individual recovery following each game, research suggests that certain performance characteristics could be used to prospectively predict individual recovery in rugby union [3, 5, 6] and other team sports [12–14]. From the present study it has been demonstrated that muscle damage, as indicated by CK concentrations following professional rugby union, is to some extent dependent on the number of physical contacts for all players, and the extent of high-speed running for backs. Therefore, despite being involved in the same team, the extent of muscle damage and subsequent recovery for one player may vary greatly from another player based on their performance characteristics. Indeed, as found by West et al. [7], the present study demonstrates a highly individual nature of recovery; with a large range in ΔCK at 40 h post-match (30 to 1849 IU.L^-1^). Thus it is proposed knowledge of individual recovery could be used to benefit player management through individual modification of training and recovery strategies. Future research to establish which recovery modalities may be more appropriate for each mechanism of muscle damage may further enhance provision post-match.

## Conclusions

This study is the first to assess the relationship between the biochemical responses to high-speed running and physical contacts using GPS technologies and performance analysis methods. The findings of the study suggest that the factors associated with muscle damage induced from match play are positional dependent. As demonstrated by previous performance analysis studies, the extent of muscle damage post-match is to some extent dependent on the total number of physical contacts induced during performance for all positions. Furthermore, the study is the first to show that for back players, muscle damage may also be due to the volume of high-speed running performed.

With previous studies demonstrating that recovery is idiosyncratic, the findings of the study suggest that GPS and performance analysis may be used by coaches and practitioners to prospectively tailor individual recovery strategies and subsequent training following match play. Thus GPS and performance analysis may have great implications for player management in rugby union.

## Abbreviations

GPS: General positioning system
CK: Creatine kinase
SSC: Stretch shortening cycle.

## References

[1] Cahill N, Lamb K, Worsfold P, Headey R, Murray S (2013). The movement characteristics of English Premiership rugby union players. J Sport Sci.

[2] Roberts SP, Trewartha G, Higgitt RJ, El-Abd J, Stokes KA (2008). The physical demands of elite English rugby union. J Sport Sci.

[3] Cunniffe B, Hore AJ, Whitcombe DM, Jones KP, Baker JS, Davies B (2010). Time course of changes in immuneoendocrine markers following an international rugby game. Eur J Appl Physiol.

[4] Quarrie KL, Hopkins WG, Anthony MJ, Gill ND (2013). Positional demands of international rugby union: evaluation of player actions and movements. J Sci Med Sport.

[5] Smart DJ, Gill ND, Beaven CM, Cook CJ, Blazevich AJ (2008). The relationship between changes in interstitial creatine kinase and game-related impacts in rugby union. Brit J Sport Med.

[6] Takarada Y (2003). Evaluation of muscle damage after a rugby match with special reference to tackle plays. Brit J Sport Med.

[7] West DJ, Finn CV, Cunningham DJ, Shearer DA, Jones MR, Harrington BJ, Crewther BT, Cook CJ, Kilduff LP (2014). Neuromuscular function, hormonal, and mood responses to a professional rugby union match. J Strength Cond Res.

[8] Elloumi M, Maso F, Michaux O, Robert A, Lac G (2003). Behaviour of saliva cortisol [C], testosterone [T] and the T/C ratio during a rugby match and during the post-competition recovery days. Eur J Appl Physiol.

[9] McLean BD, Coutts AJ, Kelly V, McGuigan MR, Cormack SJ (2010). Neuromuscular, endocrine, and perceptual fatigue responses during different length between-match microcycles in professional rugby league players. Int J Sport Physiol.

[10] Lazarim FL, Antunes-Neto JMF, da Silva FOC, Nunes LAS, Bassini-Cameron A, Cameron LC, Alves AA, Brenzikofer R, de Macedo DV (2009). The upper values of plasma creatine kinase of professional soccer players during the Brazilian National Championship. J Sci Med Sport.

[11] Kellmann M (2010). Preventing overtraining in athletes in high-intensity sports and stress/recovery monitoring. Scand J Med Sci Spor.

[12] Thorpe R, Sunderland C (2012). Muscle damage, endocrine, and immune marker response to a soccer match. J Strength Cond Res.

[13] McLellan CP, Lovell DI (2012). Neuromuscular responses to impact and collision during elite rugby league match play. J Strength Cond Res.

[14] McLellan CP, Lovell DI, Gass GC (2011). Biochemical and endocrine responses to impact and collision during elite rugby league match play. J Strength Cond Res.

[15] Nicol CK, Paavo V, Komi PV (2003). Stretch-shortening fatigue and its influence on force and power production. Strength and Power in Sport.

[16] Young WB, Hepner J, Robbins DW (2012). Movement demands in Australian rules football as indicators of muscle damage. J Strength Cond Res.

[17] Duffield R, Murphy A, Snape A, Minett GM, Skein M (2012). Post-match changes in neuromuscular function and the relationship to match demands in amateur rugby league matches. J Sci Med Sport.

[18] McLellan CP, Lovell DI, Gass GC (2011). Performance analysis of elite rugby league match play using global positioning systems. J Strength Cond Res.

[19] Gabbett TJ, Jenkins DG, Abernethy B (2012). Physical demands of professional rugby league training and competition using microtechnology. J Sci Med Sport.

[20] Gabbett TJ, Jenkins DG, Abernethy B (2011). Physical collisions and injury in professional rugby league match-play. J Sci Med Sport.

[21] Field A (2005). Discovering Statistics Using SPSS.

[22] Hopkins WG, Marshall SW, Batterham AM, Hanin J (2009). Progressive statistics for studies in sports medicine and exercise science. Med Sci Sport Exer.

[23] Cheung K, Hume PA, Maxwell L (2003). Delayed onset muscle soreness - treatment strategies and performance factors. Sports Med.

[24] Stauber WT (2004). Factors involved in strain-induced injury in skeletal muscles and outcomes of prolonged exposures. J Electromyogr Kines.

[25] Avela J, Kyrolainen H, Komi PV, Rama D (1999). Reduced reflex sensitivity persists several days after long-lasting stretch-shortening cycle exercise. J Appl Physiol.

[26] Horita T (2000). Stiffness Regulation During Stretch-Shortening Cycle Exercise.

[27] Dwyer DB, Gabbett TJ (2012). Global positioning system data analysis: velocity ranges and a new definition of sprinting for field sport athletes. J Strength Cond Res.

[28] West DJ, Cook CJ, Stokes KA, Atkinson P, Drawer SB, Bracken RM, Kilduff LP (2013). Profiling the time-course changes in neuromuscular function and muscle damage over two consecutive tournament stages in elite rugby sevens players. J Sci Med Sport.

[CR29] The pre-publication history for this paper can be accessed here:http://www.biomedcentral.com/2052-1847/6/38/prepub

